# Exploring Transcriptomic Microsatellite Markers in *Hydrangea petiolaris* (Hydrangeaceae): A Resource for Population Genetics and Functional Genomic Insights

**DOI:** 10.1002/ece3.73278

**Published:** 2026-03-16

**Authors:** Seongjun Park, Sumi Choi, Saeyeon Hwang, Eun‐Mi Lee, SeonJoo Park

**Affiliations:** ^1^ Institute of Natural Science Yeungnam University Gyeongsan Gyeongbuk South Korea; ^2^ Department of Life Sciences Yeungnam University Gyeongsan Gyeongbuk South Korea

**Keywords:** cytoplasmic male sterility, fertile and sterile flowers, *Hydrangea*, transcriptomic SSR marker

## Abstract

*Hydrangea petiolaris* Siebold & Zucc., commonly known as climbing hydrangea, is a vine native to the woodlands of Korea, Japan, and Sakhalin Island. It is an economically important ornamental species characterized by both fertile and sterile flowers. Despite growing interest in *Hydrangea* breeding and germplasm conservation, genomic resources and informative molecular markers remain limited for *H. petiolaris* , constraining population genetic and functional studies. To address this, we employed Illumina NovaSeq 6000 sequencing to generate a total of 39,945,480 RNA‐seq reads, which were assembled into 137,715 contigs. From 109,092 filtered transcripts, we identified 54,587 microsatellite repeat motifs across 33,556 contigs. Among these, 16,739 microsatellite‐containing transcripts were functionally annotated using Gene Ontology analysis, highlighting gene families such as those encoding pentatricopeptide repeat proteins, aldehyde dehydrogenases, and basic helix‐loop‐helix= transcription factors, which have been implicated in fertility restoration and floral development in plants. To develop molecular markers, we designed 45,073 primer pairs targeting microsatellite‐containing transcripts. Of the 50 tested primer pairs, 41 successfully amplified across 52 individuals, and genotyping yielded 30 polymorphic microsatellite loci with an average of 4.13 alleles per locus (ranging from 2 to 8). Two loci (*HAN33* and *HAN35*) showed evidence of null alleles and significant deviations from Hardy–Weinberg equilibrium and were treated with caution in downstream analyses. Using validated loci, population genetic analyses of two island populations (Jeju‐do and Ulleungdo) revealed strong genetic differentiation and clear clustering, illustrating the utility of these transcriptomic SSR markers for population‐level analyses and conservation‐oriented studies in *H. petiolaris* . Overall, this study provides a large genic SSR resource and an experimentally validated marker set that can be extended to broader sampling and applied to related *Hydrangea* taxa.

## Graphical abstract

This study analyzed the genetic population structure of Hydrangea petiolaris using 28 SSR markers. Distinct genetic clusters were identified, providing valuable insights for the evolution and conservation. Specifically, STRUCTURE analysis revealed clear genetic differentiation, contributing to our understanding of the species' genetic diversity.

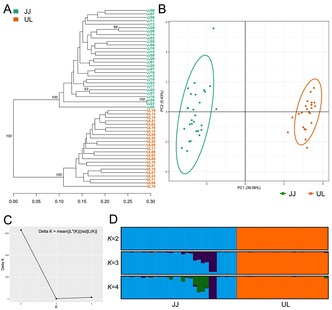

## 1 Introduction

*Hydrangea petiolaris* Siebold & Zucc., also known as climbing hydrangea, is a vine native to the woodlands of Korea, Japan, and Sakhalin Island (Iwatsuki et al. 2001). This species is sometimes considered a subspecies of the closely related *H. anomala* (McClintock 1957). It is an economically important ornamental plant that has both fertile and sterile flowers (Darwin 1897). The coexistence of these floral types raises intriguing questions about the genetic mechanisms that regulate floral diversity (Wong Sato and Kato 2019; Lu et al. 2017). In particular, understanding how fertile flowers contribute to reproductive success and the genetic structure of *H. petiolaris* will help clarify the genetic basis of floral polymorphism and its ecological and evolutionary importance. Many cultivated *Hydrangea* varieties are the result of breeding for specific traits, such as flower color, size, the timing of flower, inflorescence type, and growth habit (Cai et al. 2015; Uemachi and Okumura 2012; Adkins and Dirr 2003; Wu and Alexander 2020). These cultivars also feature distinct leaf characteristics and are frequently developed to improve adaptability and disease resistance and for other desirable qualities (Mmbaga et al. 2012; Rinehart et al. 2006). To support breeding and conservation efforts and to evaluate genetic relationships within and among wild populations, robust and transferable molecular markers are needed.

Microsatellites, also known as simple sequence repeats (SSRs), consist of one to six nucleotide repeat sequences, typically between five and forty repeats in length (Powell et al. 1996; Ellegren 2004). Because these markers are codominant, numerous, and specific to particular genomic loci, they are useful for population genetic assessments of breeding systems, genetic diversity, and conservation genetics (Slate and Pemberton 2002). Multiple polymorphic genomic SSR markers have been developed by traditional approaches and applied to genetic diversity studies in several *Hydrangea* species (Rinehart et al. 2006; Reed and Rinehart 2009, 2007); however, there is a need to identify additional genomic resources and genetic markers to explore the genetic variation within *H. anomala* and *H. petiolaris* . In addition, traditional approaches for microsatellite marker identification are often labor intensive, time consuming, and costly (Varshney et al. 2005).

Recent advances in next‐generation sequencing (NGS) have provided a faster and more cost‐effective method for discovering microsatellite markers in nonmodel plants (Ekblom and Galindo 2011; Zalapa et al. 2012). These microsatellites, which have different characteristics, can be found in genomic and transcriptome sequences. Protein‐coding genes can be linked to genomic SSRs, whereas transcriptome SSRs can be linked to protein‐coding genes and their untranslated regions (UTRs) (Varshney et al. 2005). Genomic SSRs are highly polymorphic and randomly distributed throughout the genome, whereas transcriptomic SSRs are less polymorphic and occur more frequently (Varshney et al. 2005). Transcriptomic SSRs, on the other hand, help understand relationships with functional genes and phenotypic variation, and they are transferable between closely related species, thus increasing their potential importance in generating genetic diversity for adaptive evolution (Kashi and King 2006).

The development of microsatellites is important for revealing the genetic intricacies of fertile and sterile flowers in *H. petiolaris* . Highly polymorphic microsatellites provide a robust means of assessing genetic diversity within populations, which helps us understand population structure and dynamics (Ellegren 2004; Goldstein et al. 1995). These molecular markers can be used to elucidate the reproductive strategies of *H. petiolaris* by revealing patterns of gene flow, mating systems, and reproductive success among individuals with different types of flowers. In addition, microsatellites linked to functional genes associated with fertility are valuable for identifying key genes involved in flower development (Horn et al. 2019; Li et al. 2011; El‐Namaky et al. 2016). As molecular tools, microsatellites contribute to conservation and breeding efforts by guiding the selection of individuals with desirable traits, thereby ultimately increasing the resilience and adaptability of plant populations (Rafalski and Tingey 1993; Weising et al. 1997). By studying intraspecific variation and population dynamics, microsatellites can be used to elucidate the evolutionary processes that shape the diversity of fertile and sterile flowers. Overall, the development and analysis of microsatellites provides a comprehensive approach for investigating the genetic basis of floral traits in *H. petiolaris* , with implications for basic research and applied plant biology.

In this study, we aimed to develop transcriptome‐derived SSR resources for *Hydrangea petiolaris* by assembling a leaf transcriptome and characterizing SSR abundance and distribution, functionally annotating SSR‐containing transcripts to highlight candidate genes potentially relevant to reproductive and floral traits, and designing SSR primers for marker development. We further conducted initial marker validation using two populations to assess amplification success and polymorphism and to demonstrate downstream population‐genetic applications of the validated loci.

## 2 Materials and Methods

### 2.1 Sample Collection, RNA Extraction, and Sequencing

Leaf samples were collected from a single *H. petiolaris* individual in Ulleungdo, South Korea, and a voucher specimen was deposited in the YNUH Herbarium (YNUH0HYD010 identified by SeonJoo Park). RNA was extracted from fresh leaf tissue using the HiGene Total RNA Prep Kit (BIOFACT, Daejeon, South Korea). Subsequently, 29.7 μg of *H. petiolaris* RNA was sequenced on the Illumina NovaSeq 6000 platform, resulting in 7 Gb of 150 bp paired‐end (PE) reads.

### 2.2 Transcriptome Assembly and Microsatellite Identification

The PE reads were subjected to error correction using Rcorrector v1.0.4 (Song and Florea 2015). Trinity v2.13.2 (Grabherr et al. 2011) software was used to assemble the corrected reads with the “trimmomatic” option on a 32‐core 2.6 GHz Linux Workstation with 1024 GB of memory. Next, we applied TransRate v1.0.3 (Smith‐Unna et al. 2016) to eliminate “bad transcripts”, which included chimeras, structural errors, incomplete assemblies, and base errors. Finally, we selected the microsatellite‐containing transcripts by excluding the low‐expression transcripts because the quality‐trimmed reads from Trinity were mapped to the assembled contigs to filter out low‐expression transcripts using RSEM v1.2.15 with a minimum expression (transcripts per million transcripts [TPM] matrix) of 1.0. Subsequently, these assembled contigs were utilized to identify microsatellite loci using the MIcroSAtellite identification tool (MISA) (Thiel et al. 2003). The criteria for detection included mononucleotide repeats with a minimum of 12 repeats, dinucleotide repeats with at least six repetitions, and trinucleotide and tetranucleotide repeats with a minimum of five motifs. At least four repeats of the penta‐ and hexanucleotide repeat motifs were required for detection. Compound microsatellites were identified when the interval between two repeat motifs was less than 100 nt. To explore the distribution of microsatellites in the transcriptome of *H. petiolaris* , we employed TransDecoder v5.5.0 (http://transdecoder.github.io) to identify candidate coding regions within the transcript sequences. The microsatellite locations were determined based on the predicted coding sequence (CDS) region, 5′ UTR, and 3′ UTR. We further characterized and compared motif types among microsatellite loci in the CDS region, 5′ UTR, and 3′ UTR. We adjusted significance levels for multiple comparisons using the false discovery rate. Statistical analyses were performed using R v4.1.2 (Team 2021).

### 2.3 Functional Annotation

To understand the possible functions of microsatellites, we searched for microsatellite‐containing transcripts in the GenBank nonredundant (nr) protein database. This search was performed using BLASTP with an *e*‐value threshold of 10^−5^ using Blast2GO v4.1.9 (Götz et al. 2008).

### 2.4 Microsatellite Detection and Genotyping

To assess the amplification and polymorphism of microsatellite loci identified in the *H. petiolaris* leaf transcriptome, we designed primers based on sequences flanking these loci using Primer3 (Untergasser et al. 2012). From the filtered transcripts with different repeat sizes, 50 primer pairs were selected for initial PCR screening. Candidates were prioritized by longer repeat arrays within each motif class (excluding mononucleotide repeats), Gene Ontology (GO)‐annotated transcripts, and loci with sufficient flanking sequence for primer design. Total genomic DNA was extracted from 52 individuals collected from Jeju‐do (JJ, *n* = 29) and Ulleungdo, Gyeongbuk (UL, *n* = 23), South Korea, which served as the initial validation set to assess cross‐population amplification and polymorphism of the developed SSR loci. Polymerase chain reaction (PCR) was performed in a 25‐μL reaction mixture containing 10 ng of genomic DNA, each dNTP at 0.2 mM, each primer at 0.25 μM, 5 μL of 10× *h*‐Taq reaction buffer, and 0.25 units of *h*‐Taq polymerase (Solgent Co., Daejeon, South Korea). The PCR procedure included initial denaturation at 95°C for 15 min; 30 cycles of 95°C for 20 s, 58°C or 60°C for 40 s, and 72°C for 30 s; and a final extension at 72°C for 5 min. Electrophoresis on a 2% agarose gel was used to confirm amplification and to assess polymorphisms. For fragment analyses, forward or reverse primers (Table 1) were labeled with fluorescent dyes (6‐FAM, HEX, or TAMRA). PCR products amplified with these primers were subsequently genotyped using an ABI 3730xl DNA Analyzer at Macrogen Inc. (Seoul, South Korea).

**TABLE 1:** Primer sequences and characterization of the 30 microsatellite loci developed for *Hydrangea petiolaris*.

Locus	Primer sequence (5′‐3′)	Repeat motif	SSR distribution	BLAST
*HAN06*	TTGTTTGGGATCTCACATTCTCCA	(CT)n	CDS	NAC domain‐containing protein 21/22
	ATGTCCCAAGGTTCGCACTT			
*HAN08*	AGTCCCACACCAATACCCCT	(TCA)n	CDS	GEM‐like protein 5
	AGGCCTTTGGGTTATTTGGG			
*HAN09*	TGTTTTGTGCCCCTCTTTGG	(TCT)n	CDS	StAR‐related lipid transfer protein 7, mitochondrial
	AACCCAAGAAATGCAGCTGG			
*HAN10*	GCTCTCCTCCATAAACCCTC	(GAA)n	5′UTR	mRNA adenosine methylase
	CCAGCTGTTGACGCATATCC			
*HAN13*	CTTTCTCTGATCTCGCCGCA	(TAT)n	5′UTR	Syndetin‐like
	GTTCCCTCGAACCTACCACC			
*HAN14*	ATGAAACCACCCCAGTGTCC	(AAG)n	CDS	Transcription factor MYB18
	TTCCTTGGGACTCAGAGCCT			
*HAN16*	GCGGCCTGTTTATGAAACCG	(TCT)n	3′UTR	Putative histone deacetylase
	GCACAAGAGCTGACATTACACAG			
*HAN17*	TCAGCACAGCCACCAAAATC	(TCT)n	CDS	Organelle RRM domain‐containing protein 1, chloroplastic‐like
	TGGCCACTGGAAATGGAAGG			
*HAN19*	TTGCCAATGTCCTCCTCTCT	(TAT)n	CDS	Putative 2‐aminoethanethiol dioxygenase
	AGCAAGTGGGGTTCCTGAAG			
*HAN20*	ACACCAGAGTTCACAGGCTG	(CCA)n	CDS	RING‐H2 finger protein ATL54
	TGGAGAAAGGGAGAGCGAGT			
*HAN21*	CGACCTCTCCAAACCTGACC	(GAA)n	CDS	RNA polymerase II transcriptional coactivator KELP
	CGCCGTCGTCATTGTACTCT			
*HAN22*	CATGATGAGCAATCGCCGTC	(GAT)n	CDS	E3 ubiquitin‐protein ligase SIRP1‐like isoform X2
	CTGGCCGAACTTCTCCTTGT			
*HAN23*	TGGATGAAGCAATCCCACAA	(ACG)n	CDS	PlSAUR4 protein
	AGTTTCGCTATGTCCAGGCC			
*HAN24*	CAACCTCTAACCCGACCACC	(GAA)n	CDS	Basic helix–loop–helix transcription factor
	CCGTTACTACGTCCTGAGCC			
*HAN25*	TTCTTCTGCTCAGTCACGCG	(ATA)n	CDS	Pentatricopeptide repeat‐containing protein
	TGGTTACGCCTCAACAAGCT			
*HAN28*	TTGAAGAACAGGCGGTCGAA	(CCA)n	CDS	Leucine‐rich repeat receptor‐like serine/threonine‐protein kinase BAM2
	GCTAAGAAGATCCGGCAGCA			
*HAN31*	GGCAAAACCCCTCGCATTTT	(TCT)n	CDS	Transcription initiation factor TFIID subunit 12 isoform X2
	GACCCAACACACCAAACTGC			
*HAN32*	AATTGGGTTTAAGGGGGCCC	(GAA)n	5′UTR	Mitochondrial phosphate carrier protein 1, mitochondrial‐like
	TGGGTTGTTCCAGCACTGAG			
*HAN33*	TGGGGTTAGGGTTAGGGTTT	(CAA)n	5′UTR	Serine/threonine‐protein phosphatase PP2A‐4 catalytic subunit
	GAGGTTGCCATGTGTGTTCG			
*HAN34*	ATCTCGCCGCACTCTCAAAA	(GAA)n	5′UTR	Syndetin‐like
	CCTCGCTCAAATCGCCATTG			
*HAN35*	AACGCCATTCCTCTGCAAAA	(CCA)n	CDS	DNA mismatch repair protein MutS, type 2
	TGACTGACCTACTACGGCCA			
*HAN36*	CCCTCCACCTCCACTCTTCA	(TCA)n	CDS	Rubisco accumulation factor 1.1, chloroplastic‐like
	GTTGGCAAGGATTTCGAGGC			
*HAN37*	GCGATCGGACCATGATGACT	(CAA)n	CDS	Pentatricopeptide repeat‐containing protein At1g77360, mitochondrial‐like
	TGGCTATGATTTCGCAGAGT			
*HAN38*	CCTTGCTCTTTCAGGTACCC	(CTTAA)n	CDS/3′UTR	Homeobox‐leucine zipper protein ATHB‐12
	GGAGCGGAACCCTTTTAACT			
*HAN40*	AAACCCACCACCATCACCTC	(CCTCAG)n	CDS	Histone H1‐like
	CACAAAGACCGACTCTGGCT			
*HAN41*	GCAATTGTGTACTTCCGGCG	(AACCCT)n	CDS	DeSI‐like protein
	TGGTGCAGATGGAGGAAAGC			
*HAN42*	AGGCGACACTAACCACTTCG	(CCTAAC)n	CDS	Glutaredoxin protein
	GGCCTTCCATGAGAACCCAA			
*HAN44*	GGAAAAGGACCGAGTACCCA	(GAGAAA)n	CDS	SART‐1 family protein DOT2‐like isoform X1
	CGTTATCTTGTCCCCGCTCA			
*HAN46*	TCCACAACGTGAAAACCCCA	(CAGAGT)n	CDS	Pentatricopeptide repeat‐containing protein At2g15690, mitochondrial‐like
	CCCTCTCGGCACAAACTCAT			
*HAN47*	CTCCTTCATCACCACCGTCA	(CCACAG)n	CDS	Zinc‐finger homeodomain protein 6‐like
	TCCCCGCCTGACATGAATTC			

### 2.5 Population Genetic Diversity and Structure Analyses

Electropherograms were inspected and allele sizes were scored in Geneious Prime 2023.2.1 (www.geneious.com). Null‐allele frequencies were estimated using FreeNA (Chapuis and Estoup 2007). MICRO‐CHECKER v2.2.3 (Van Oosterhout et al. 2004) was used to detect genotyping errors, including null alleles. Microsatellite polymorphisms in *H. petiolaris* were evaluated based on several genetic parameters, including the average number of alleles per locus (*N*_A_), observed (*H*_O_) and expected (*H*_E_) heterozygosity, and inbreeding coefficient (*F*_IS_) (Weir and Cockerham 1984). This analysis was performed using Arlequin v3.5.2.2 (Excoffier and Lischer 2010) and FSTAT v2.9.3.2 (Goudet 2002). Analysis of molecular variance (AMOVA) and population differentiation (*F*_ST_) were estimated in Arlequin. Departures from Hardy–Weinberg equilibrium (HWE) were tested in GENEPOP v4.8.5 (Raymond 1995) using exact tests based on the Markov chain method (Guo and Thompson 1992). Linkage disequilibrium (LD) between loci was evaluated in GENEPOP using exact‐test based on the Markov chain method, and significance levels were adjusted using Bonferroni correction for multiple comparisons. We calculated the polymorphic information content (PIC) using CERVUS v3.0.7 (Kalinowski et al. 2007). STRUCTURE v2.3.4 (Pritchard et al. 2000) was utilized to determine the genetic makeup of the population and to evaluate variations among different populations. The range of clusters (*K*) was defined as 1– 5, and the burn‐in period and Markov Chain Monte Carlo (MCMC) were set at 10,000 and 100,000, respectively. To ensure the accuracy of the results, each run was replicated 10 times. The output results of STRUCTURE were visualized using the Cluster Markov Packager Across K (CLUMPAK) program (Kopelman et al. 2015). Principal component analysis (PCA) and the unweighted pair group method with arithmetic means (UPGMA) were also performed using the *ade4* and *poppr* packages in R, respectively.

## 3 Results

### 3.1 Characteristics of Microsatellites in the *Hydrangea petiolaris* Transcriptome

We assembled 39,945,480 reads from RNA‐seq into 137,715 contigs, each averaging 885 bp (Table S1–S51). We then excluded transcripts that had structural and base errors and transcripts with low expression levels by TransRate and RSEM analyses (Table S1–S51). Among the 109,092 filtered transcripts, 33,556 had 54,587 microsatellite repeat patterns, representing 30.8% of the dataset (Table S2). Among these SSRs, dinucleotide microsatellites were the most abundant (62.2%), followed by trinucleotide (10.7%), hexanucleotide (2.8%), pentanucleotide (1.6%), and tetranucleotide (1.1%) motifs (Figure S1). Among the mononucleotide repeats, A/T motifs were dominant, accounting for 96% of the total. For dinucleotide motifs, AG/CT was the most common type, accounting for 57.2% of the occurrences, while CG/GC occurred only once (Figure S1). Ten different trinucleotide motifs were identified, of which AAG/CTT was the most common (approximately 31%), while CCG/CGG accounted for only 3% of the occurrences (Figure S1). The average length of microsatellites in the *H. petiolaris* transcriptome was 18.96 bp (Figure S2). Differences in length between each motif size class were statistically significant (pairwise Wilcoxon rank sum test, *p* < 2 × 10^−16^ after Bonferroni correction), except for tetra‐ and pentanucleotide motifs (Figure S2). The mononucleotide motifs had the shortest average length of 16.7 bp, while the hexanucleotide motifs had the longest average length (27.4 bp). The longest microsatellite identified was 74 bp in length, with a 37‐fold repetition of a dinucleotide motif (Figure S2).

**FIGURE 1:**
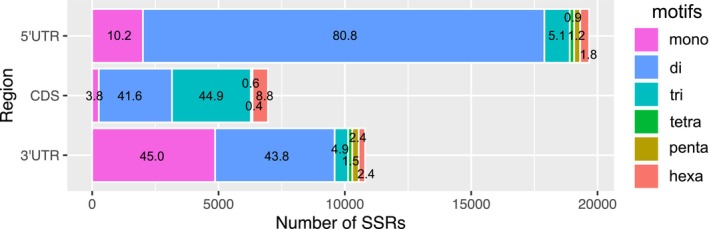
Frequencies of SSRs in different genic regions.

The distributions of microsatellites in the 5′ UTR, CDS region, and 3′ UTR were investigated. Among 54,587 microsatellites, 19,666 were found in the 5′ UTR, 6957 in the CDS region and 10,802 in the 3′ UTR (Table S3). The remaining 17,162 microsatellites were eliminated because the information in the transcripts needed to determine the CDS region was lacking. The frequencies of microsatellites in the CDS region were lower than those in the UTRs (Table S3). The location had a significant impact on the frequency of motif size classes (Kruskal–Wallis rank sum test, *p* < 2.2 × 10^−16^). For example, a significant proportion of the microsatellites found in the CDS region were trinucleotide motifs (44.9%). The UTRs were mostly composed of mono‐ and dinucleotide microsatellites, with mono‐ (mean 22.6%) and dinucleotide (mean 67.7%) motifs being much more abundant in the UTRs than in the CDS region (Figure 1). The average length of the microsatellites varied significantly between the CDS regions and the 3′ UTR and between the 5′ UTR and the 3′ UTR (Kruskal–Wallis rank sum test, *p* < 2 × 10^−16^; Figure S3). The effect of location on the average length of the four motif size classes was statistically significant (Kruskal–Wallis rank sum test, mono‐: *p* = 0.03192, di‐: *p* = 5.769 × 10^−8^, tri‐: *p* < 2.2 × 10^−16^, hexa‐: *p* = 0.0187; Figure S3).

### 3.2 Functional Annotation of Genes Containing Microsatellites

**FIGURE 2:**
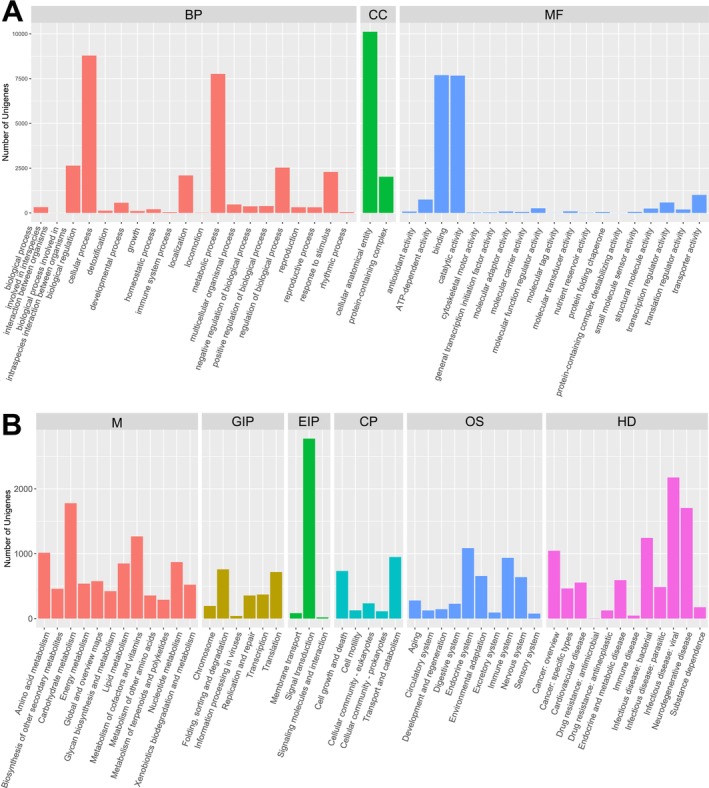
Functional annotation analysis of genes containing microsatellites. (A) Classification of genes containing microsatellite loci based on the Gene Ontology (GO) annotation. (B) KEGG pathways associated with genes containing microsatellites.

The microsatellite‐containing transcripts were categorized based on their functions using GO analysis. For GO mapping and annotations, 23,007 transcripts (excluding mononucleotide repeats) were selected, resulting in 27,006 ORFs. The amino acid sequences of the 27,006 ORFs were subjected to a BLAST search, and 19,590 (72.54%) of the sequences were found to match “Viridiplantae (taxonomy ID: 33090)” in the nr protein database. GO annotations were obtained for 16,739 (61.98%) microsatellite‐containing transcripts, which were classified into three functional groups with 41 subgroups (Figure 2A). In the “biological process” category, the most prominent subgroups were cellular (8788) and metabolic processes (7764). Within the “molecular function” group, binding (7695) and catalytic activity (7668) contained the greatest number of genes. Within the “cellular component” group, the cellular anatomical entity (10,118) and protein‐containing complex (2021) had the most enrichment of genes. We identified numerous transcripts as pentatricopeptide repeat (PPR) proteins, aldehyde dehydrogenases, acyl carrier proteins, glycine‐rich proteins, peptidase‐like proteins, and basic helix–loop–helix (bHLH) transcription factors (Table S4). KEGG pathway analysis was used to further explore the functions of microsatellite‐containing transcripts and the results revealed that 7663 transcripts were involved in 421 pathways (Figure 2B and Table S5). These KEGG pathways were grouped into six functional categories, with metabolism (34.9%), human diseases (12.9%), organismal systems (6.8%), and environmental information processing (1.5%) being the most prominent (Figure 2B). The notable pathways included *plant hormone signal transduction* (ko04075), *meiosis‐yeast* (ko04113), *DNA replication* (ko03030), *MAPK signaling pathway—plant* (ko04016), and *ubiquitin‐mediated proteolysis* (ko04120) (Table S5).

### 3.3 Validation of Transcriptomic SSR Markers

The primers used to amplify the 45,073 microsatellites were designed based on 54,587 transcripts containing microsatellites. We selected a total of 50 primer pairs based on the following criteria: (1) prioritizing microsatellites with longer repeat motifs and excluding mononucleotide repeats and (2) a filtering step to exclude low‐expression transcripts to ensure the selection of robust primer pairs for amplification and polymorphism detection. Four primer pairs did not yield amplicons, but 46 pairs were amplified consistently and successfully from 52 individuals of the JJ (Jeju‐do) and UL (Ulleungdo) populations. Of the 46 primer pairs, 41 produced amplicons of the expected size, while the rest produced amplicons larger than the expected size. We identified 30 polymorphic microsatellite loci and successfully genotyped them in 52 individuals (Table 1). The 30 identified microsatellite loci had GO annotations (Table 1).

The average number of alleles per SSR locus was 4.13 (Table 2). The estimated null allele frequency of *HAN09*, *HAN33*, *HAN35*, and *HAN38* was greater than 0.1 (Table 2), and MICRO‐CHECKER also indicated a greater frequency of null alleles at two loci (*HAN33* and *HAN35*). The results showed no significant deviation from HWE except for two loci (*HAN33* and *HAN35*) after correction based on the false discovery rate (*p* < 0.05). A LD test indicated that most loci were in linkage equilibrium, significant associations detected for four pairs of loci (*HAN06* and *HAN36*, *HAN10* and *HAN36*, *HAN13* and *HAN34*, and *HAN25* and *HAN35*) (Figure S4). The observed (*H*_O_) and expected (*H*_E_) heterozygosities ranged from 0.1379 to 0.8276 and 0.1307 to 0.7737, respectively, and the locus‐specific *F*_IS_ estimates ranged from −0.2 (*HAN37*) to 0.524 (*HAN33*) (Table 2). The PIC ranged from 0.145 (*HAN22*) to 0.726 (*HAN21*) for the 30 SSR markers (Table 2). Notably, *HAN21*, which exhibited a high PIC value, indicating substantial genetic variation, was associated with the transcriptional coactivator p15 (PC4) family protein (KELP) (Table 2).

**TABLE 2:** Summary statistics for 30 polymorphic microsatellite loci of *Hydrangea petiolaris*.

Locus	Size range (bp)	*N*_A_	*F*null	*H*_O_	*H*_E_	*F*_IS_	HWE	PIC
*HAN06*	229–255	8	0.00000	0.7931	0.74592	−0.064	0.0751	0.692
*HAN08*	211–226	5	0.00000	0.58621	0.56382	−0.040	0.1934	0.474
*HAN09*	217–241	6	0.10269	0.51724	0.69631	0.261	0.0853	0.631
*HAN10*	217–238	8	0.01329	0.68966	0.71264	0.033	0.4595	0.662
*HAN13*	264–273	4	0.04534	0.44828	0.53479	0.164	0.3571	0.445
*HAN14*	159–162	2	0.09371	0.24138	0.3539	0.322	0.1109	0.287
*HAN16*	256–280	5	0.02873	0.62069	0.7199	0.140	0.4253	0.662
*HAN17*	236–290	6	0.08707	0.58621	0.74773	0.219	0.3322	0.690
*HAN19*	204–216	3	0.00754	0.48276	0.51361	0.061	0.5612	0.394
*HAN20*	167–176	3	0.01315	0.48276	0.52692	0.085	0.8542	0.415
*HAN21*	193–211	6	0.00000	0.82759	0.77374	−0.071	0.4998	0.726
*HAN22*	212–215	2	0.00001	0.17241	0.16031	−0.077	1.0000	0.145
*HAN23*	109–124	3	0.00001	0.24138	0.22202	−0.089	1.0000	0.205
*HAN24*	239–242	2	0.00001	0.13793	0.13067	−0.057	1.0000	0.120
*HAN25*	178–181	2	0.06327	0.34483	0.43557	0.211	0.3822	0.336
*HAN28*	230–251	5	0.00000	0.68966	0.69449	0.007	0.3708	0.633
*HAN31*	274–295	6	0.00000	0.72414	0.73321	0.013	0.4137	0.672
*HAN32*	275–278	2	0.05346	0.17241	0.21597	0.205	0.3356	0.190
*HAN33*	160–184	8	0.21081	0.35714	0.74351	0.524	0.0000**	0.695
*HAN34*	236–245	4	0.04534	0.44828	0.53479	0.164	0.3571	0.445
*HAN35*	157–184	6	0.19919	0.37931	0.71748	0.476	0.0001**	0.662
*HAN36*	185–200	5	0.08344	0.55172	0.67998	0.191	0.2894	0.623
*HAN37*	238–253	2	0.00000	0.35714	0.2987	−0.200	0.5531	0.250
*HAN38*	200–205	2	0.10315	0.34483	0.50817	0.325	0.1341	0.375
*HAN40*	192–204	3	0.00011	0.44828	0.50272	0.110	0.0569	0.444
*HAN41*	218–230	3	0.00000	0.37931	0.32728	−0.162	1.0000	0.293
*HAN42*	218–230	3	0.00006	0.48276	0.49062	0.016	0.7232	0.406
*HAN44*	236–248	3	0.00000	0.62069	0.58802	−0.057	1.0000	0.492
*HAN46*	262–268	2	0.00003	0.41379	0.40653	−0.018	1.0000	0.320
*HAN47*	212–236	5	0.00001	0.51724	0.49546	−0.045	1.0000	0.431

*Note:* Significant values after false discovery rate correction are indicated with asterisks (* *p* < 0.05, **p < 0.01).

Abbreviations: *F*_IS_, inbreeding coefficient; *F*null, frequency of null allele; *H*_E_ heterozygosities expected, *H*_O_, heterozygosities observed; HWE, Hardy–Weinberg equilibrium; *N*_A_, Number of alleles; PIC, polymorphism information content.

**FIGURE 3:**
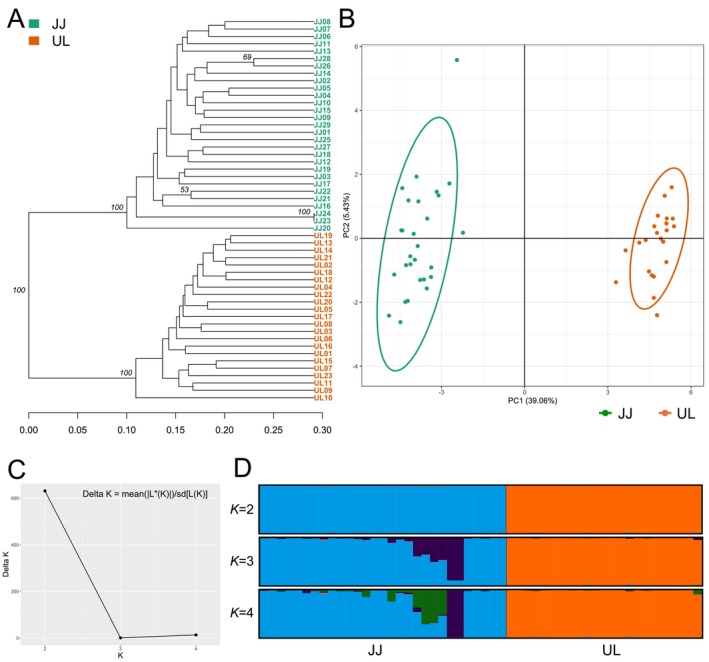
Genetic population structure of *Hydrangea petiolaris* based on 28 SSR markers. (A) Unweighted pair group method with arithmetic mean (UPGMA) tree based on Nei's genetic distance. (B) Scatterplots of principal component analysis (PCA) based on the first and two components. (C) Estimation of delta *K* with cluster number (*K*) ranged from one to five. (D) Bayesian clustering results of the STRUCTURE analysis. Genetic group structure with *K* = 2, 3 and 4.

Population‐level analyses were conducted using 28 SSR markers after excluding *HAN33* and *HAN35* due to evidence of null alleles and significant deviation from HWE. At the population level, the JJ population exhibited slightly greater genetic diversity (*H*_O_ = 0.47581, *H*_E_ = 0.51121) and a lower inbreeding coefficient (*F*_IS_ = 0.070) than the UL population (*H*_O_ = 0.42468, *H*_E_ = 0.47795, and *F*_IS_ = 0.114) (Table 3). The mean Garza‐Williamson (G‐W) indices for the JJ and UL populations were 0.3048 and 0.2479, respectively (Table 3). The AMOVA revealed 39.33% and 60.67% of genetic variation among and within populations, respectively (Table 4). The JJ and UL populations showed high genetic differentiation (*F*_ST_ = 0.39329) (Table 4). The UPGMA dendrogram, based on genetic distances between populations, showed that the wild populations of *H. petiolaris* can be divided into two distinct branches, with JJ and UL clustering separately (Figure 3A). The PCA plot showed that the two groups were clearly separated (Figure 3B). The STRUCTURE simulation confirmed that the highest peak of delta *K* was at *K* = 2 (Figure 3C). On the basis of the most likely *K* value (*K* = 2), the two populations of *H. petiolaris* were divided into two genetically distinct groups (Figure 3D). Note that when *K* = 3, the highland individuals (> 680 m) of the JJ population were further separated from the lowland individuals (< 530 m), showing admixed clusters (Figure 3D).

**TABLE 3:** Genetic diversity within two *Hydrangea petiolaris* populations based on 28 microsatellite markers.

Population	Abbreviation	*n*	t*N*_A_	m*N*_A_	*H*_O_	*H*_E_	*F*_IS_	mPIC	G‐W
Jeju‐do	JJ	29	110	3.929	0.47581	0.51121	0.070	0.4605	0.3048
Ulleungdo	UL	23	97	3.556	0.42468	0.47795	0.114	0.4008	0.2479

Abbreviations: *F*_IS_, inbreeding coefficient; G‐W, Garza‐Williamson index; *H*_E_, heterozygosities expected; *H*_O_, heterozygosities observed; m*N*_A_, Mean number of alleles; mPIC, Mean polymorphism information content; *n*, Sample size; *N*_A_, Number of alleles; t*N*_A_, Total number of alleles.

**TABLE 4:** Analysis of molecular variance (AMOVA) of two *Hydrangea petiolaris* populations based on 28 microsatellite markers.

Source of variation	Sum of squares	Variance components	Percentage variation	Fixation indices
Among populations	234.064	4.43697	39.32866	*F*_ST_ = 0.39329**
Within populations	696.693	6.84481	60.67134	
Total	930.757	11.28178		

** Significant value at *p* < 0.01, *F*_ST_: average inbreeding coefficient of subpopulations relative to the total population.

## 4 Discussion

Transcriptome sequencing has emerged as a valuable tool for identifying SSR markers, as demonstrated in numerous studies (Hodel et al. 2016; Biswas et al. 2020; Park et al. 2019). Our investigation of microsatellites in the transcriptome of *H. petiolaris* provides new insights into their abundance, distribution, and functional significance. The distribution of SSR motif classes across genic regions observed here (e.g., enrichment of trinucleotide repeats in CDS relative to UTRs) is consistent with general patterns reported for transcriptome‐derived SSR resources in non‐model plants (Varshney et al. 2005; Zalapa et al. 2012). In *Hydrangea*, previous SSR resources were primarily developed from genomic DNA and have been applied to genetic diversity studies (Rinehart et al. 2006; Reed and Rinehart 2007, 2009). Our transcriptome‐derived SSR set complements these genomic SSR markers by providing a large genic marker resource with functional annotation, together with an experimentally validated subset of polymorphic loci suitable for downstream population and conservation applications. Together, these results provide a genic marker resource for *H. petiolaris* and a starting point for linking SSR variation to annotated transcripts relevant to floral and reproductive traits (Ellegren 2004). Functional annotation of microsatellite‐containing genes identified a diverse set of biological processes, cellular components, and molecular functions, highlighting key genes encoding PPR proteins, aldehyde dehydrogenases, acyl carrier proteins, glycine‐rich proteins, peptidase‐like proteins, and bHLH transcription factors (Table S4).

These annotations highlight the diverse functional contexts of SSR‐containing transcripts, including gene families previously implicated in floral development and fertility restoration. For example, PPR proteins constitute a major gene family associated with fertility restoration in plants, and we identified SSR‐containing transcripts annotated as PPR proteins in *H. petiolaris* . These loci provide candidate functional markers for exploring cytoplasmic male sterility (CMS)‐related pathways and floral polymorphism in this species (Kim and Zhang 2018). Aldehyde dehydrogenases were also detected among SSR‐containing transcripts, consistent with their reported involvement in flower development in other plant systems (Liu et al. 2001). Similarly, acyl carrier proteins, known for their role in fatty acid biosynthesis and lipid metabolism, were detected, suggesting a possible link between lipid metabolic pathways and floral traits (Fujii and Toriyama 2009; Bonaventure et al. 2003). The presence of glycine‐rich proteins among the microsatellite‐containing transcripts in *H. petiolaris* points to their potential involvement in stress‐response pathways and floral‐trait regulation (Itabashi et al. 2011; Sachetto‐Martins et al. 2000). Additionally, peptidase‐like proteins, which are involved in protein degradation and processing (Kitazaki et al. 2015), may contribute to proteomic dynamics that regulate floral development. We also identified SSR‐containing transcripts annotated as bHLH transcription factors, a gene family widely implicated in transcriptional regulation during flower development, highlighting candidate loci for future investigations of floral‐trait variation (Jaqueth et al. 2020).

Because functional inference is based on homology searches against currently available reference databases, GO terms could be assigned to ~62% of SSR‐containing transcripts, whereas the remaining ~38% lacked significant matches, which may reflect lineage‐specific sequences, untranslated regions, or transcript fragments despite filtering.

To provide functional context for these annotated SSR‐containing transcripts beyond GO categories, we examined KEGG pathway assignments. Our results highlight the involvement of plant hormone signal transduction pathways, which regulate auxin, gibberellin, and cytokinin signaling—key processes in floral initiation, organ differentiation, and reproductive success (Fleet and Sun 2005; Sakakibara 2006; Aloni 1980). Although the meiosis‐yeast pathway is not plant‐specific, it remains critical for understanding meiotic processes essential for reproductive cell development and fertility (Mercier and Grelon 2008). DNA replication pathways further underscore the role of microsatellites in maintaining genomic stability during reproductive cell formation (Ellegren 2004; Yant et al. 2010). The representation of MAPK signaling and ubiquitin‐mediated proteolysis among annotated SSR‐containing transcripts suggests that this marker resource also captures genes involved in stress responses, development, and post‐translational regulation relevant to floral biology (Colcombet and Hirt 2008; Cristina et al. 2010; Santner and Estelle 2010). These findings deepen our understanding of microsatellite function and highlight potential avenues for further research into their role in adaptive responses in *H. petiolaris* .

Beyond their functional roles, transcriptomic SSRs provide a valuable resource for assessing genetic diversity, population structure, and evolutionary patterns. Our validation of SSR markers in *H. petiolaris* reveals important genetic characteristics of this wild species. We identified 30 polymorphic microsatellite loci, offering a detailed perspective on genetic variation. However, caution is necessary due to the presence of null alleles at loci *HAN33* and *HAN35*, which may affect genotyping accuracy. The observed deviations from HWE at these loci underscore the importance of using robust statistical approaches in population genetics analyses. LD analysis identified significant associations among four pairs of loci (Figure S4), suggesting potential genetic linkage or physical proximity. Although experimental screening was performed on a subset of primer pairs (50 tested), yielding 30 polymorphic loci, the broader primer resource generated here provides a flexible basis for expanding or tailoring marker panels for additional populations and applications. Because transcriptome‐derived SSRs are located in expressed regions, they are often transferable across closely related taxa (Zalapa et al. 2012; Varshney et al. 2005). This cross‐species applicability facilitates broader investigations into genetic diversity within the *Hydrangea* genus and related taxa.

Using the validated SSR loci, we detected clear genetic differentiation between the JJ and UL populations, demonstrating the utility of these markers for resolving population‐level patterns in *H. petiolaris* . Extending sampling across additional localities will allow these loci to be applied to broader inferences of population connectivity and geographic structure. The JJ population exhibited slightly higher genetic diversity and a lower inbreeding coefficient than the UL population, consistent with population‐specific differences in mating system and demographic history. Substantial genetic differentiation (*F*_*ST*_ = 0.3933) between the JJ and UL populations suggests limited contemporary gene flow between these two island populations, consistent with geographic separation. AMOVA results indicate that 39.33% of genetic variation is attributable to differences between populations, reinforcing the importance of conserving population‐specific genetic diversity. The UPGMA and PCA patterns are consistent with this JJ–UL separation, supporting the utility of the developed loci for population structure analyses. Future studies incorporating ecological and environmental data could provide deeper insights into the factors driving population differentiation and inform conservation efforts aimed at mitigating habitat fragmentation and climate change effects on wild populations. A comprehensive understanding of genetic diversity and structure in *H. petiolaris* will require further studies, particularly in Japanese and Sakhalin Island populations.

The functional annotations and validated markers identified in this study offer a valuable foundation for future research. Investigating the specific regulatory roles of microsatellites in gene expression could reveal new insights into their influence on molecular networks in *H. petiolaris* . Comparative genomic analyses with related plant species may help elucidate the evolutionary dynamics of microsatellites, shedding light on their conservation and divergence across taxa. The integration of genomic and transcriptomic data will provide a more comprehensive perspective on the genetic architecture of *H. petiolaris* . Additionally, applying these SSR markers in ecological and population studies may contribute to informed conservation strategies and sustainable management practices for this species.

## Author Contributions

**Seongjun Park:** conceptualization (equal), data curation (lead), formal analysis (lead), methodology (equal), visualization (lead), writing – original draft (lead), writing – review and editing (equal). **Sumi Choi:** investigation (equal), writing – review and editing (equal). **Saeyeon Hwang:** investigation (equal), writing – review and editing (equal). **Eun‐Mi Lee:** data curation (equal), writing – review and editing (equal). **SeonJoo Park:** conceptualization (equal), funding acquisition (lead), writing – review and editing (equal).

## Conflicts of Interest

The authors declare no conflicts of interest.

## Supporting information


**Figures S1–S4:** ece373278‐sup‐0001‐Figures.pdf.

**Tables S1–S51:** ece373278‐sup‐0002‐Tables.xlsx.

## Data Availability

The data that support the findings of this study are available in the Supporting Information of this article. The datasets generated during the current study are available in the Dryad Digital Repository, (https://doi.org/10.5061/dryad.7pvmcvf1n).
