# Supplementary material for: Exploring Transcriptomic Microsatellite Markers in Hydrangea petiolaris (Hydrangeaceae): A Resource for Population Genetics and Functional Genomic Insights
Source: Ecol Evol. 2026 Mar 16;16(3):e73278. doi: 10.1002/ece3.73278 (PMC13093395; doi:10.1002/ece3.73278)

Figure S1. The distributions of the major repeat types in the *Hydrangea petiolaris* leaf transcriptome.

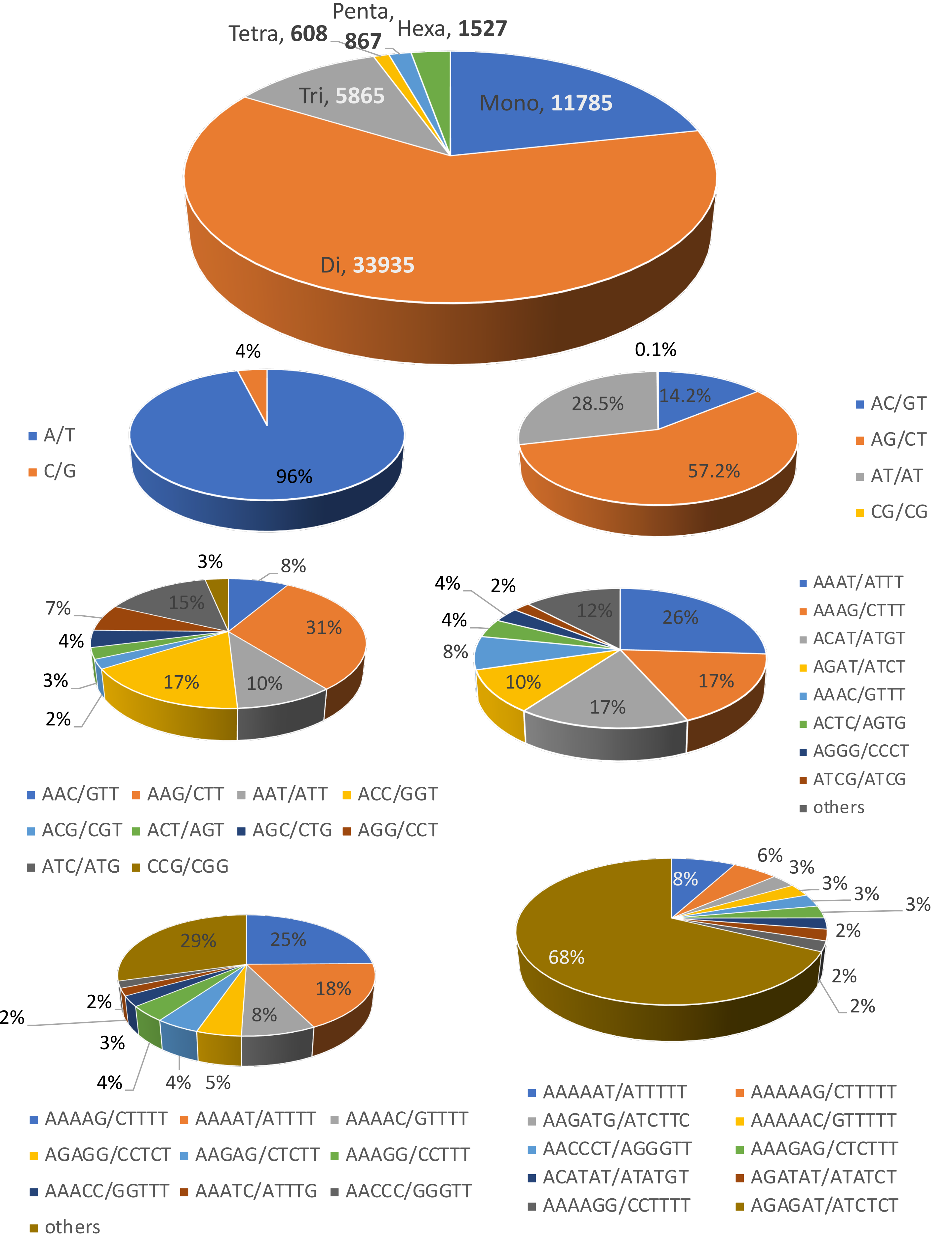

Figure S2. Box plots of the sizes of different repeat motifs. The box represents values between quartiles, solid lines extend to minimum and maximum values, outliers are shown as circles and horizontal lines in boxes show median values.

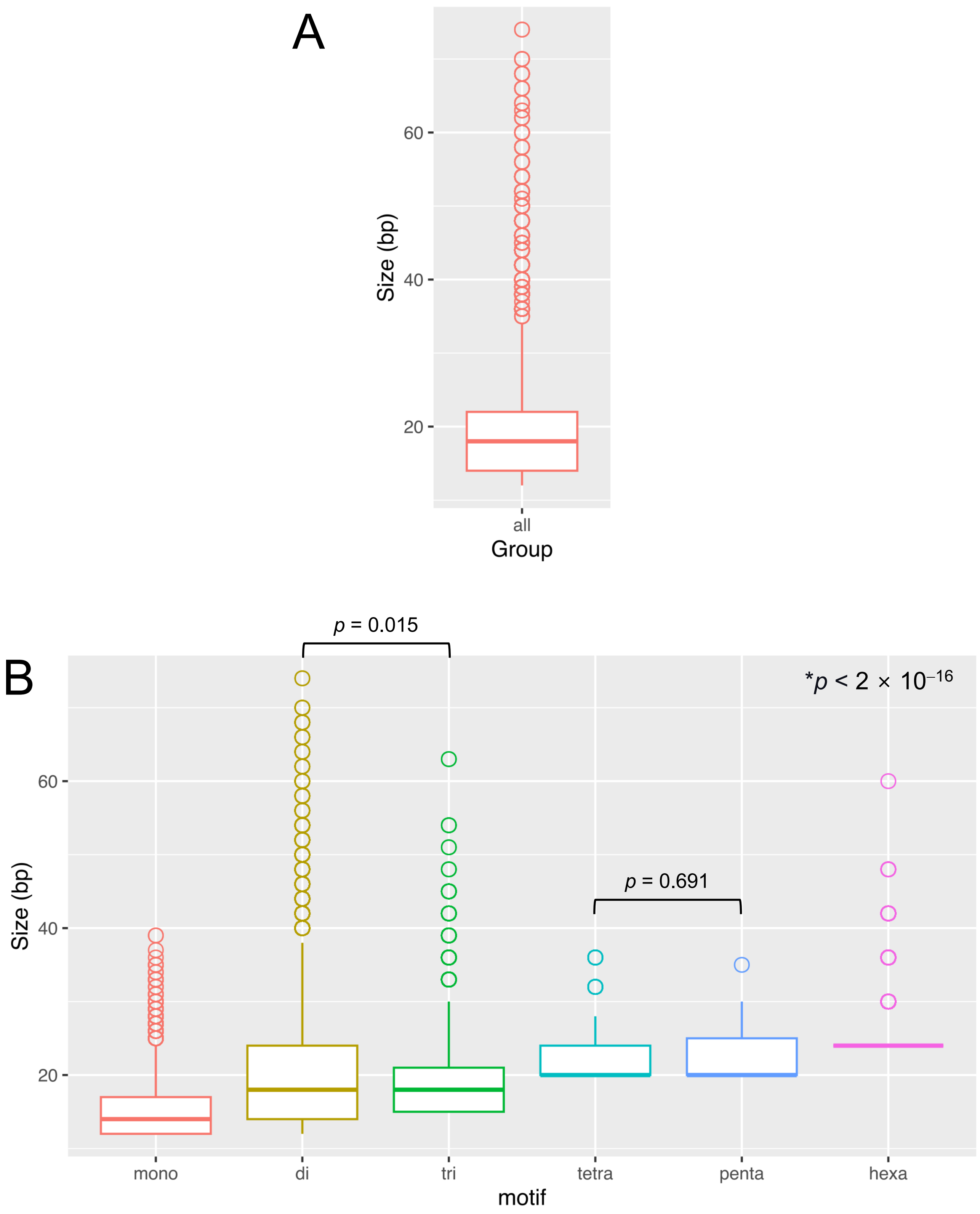

Figure S3. Box plots of the sizes of different repeat motifs in different genic regions. The box represents values between quartiles, solid lines extend to minimum and maximum values, outliers are shown as circles and horizontal lines in boxes show median values.

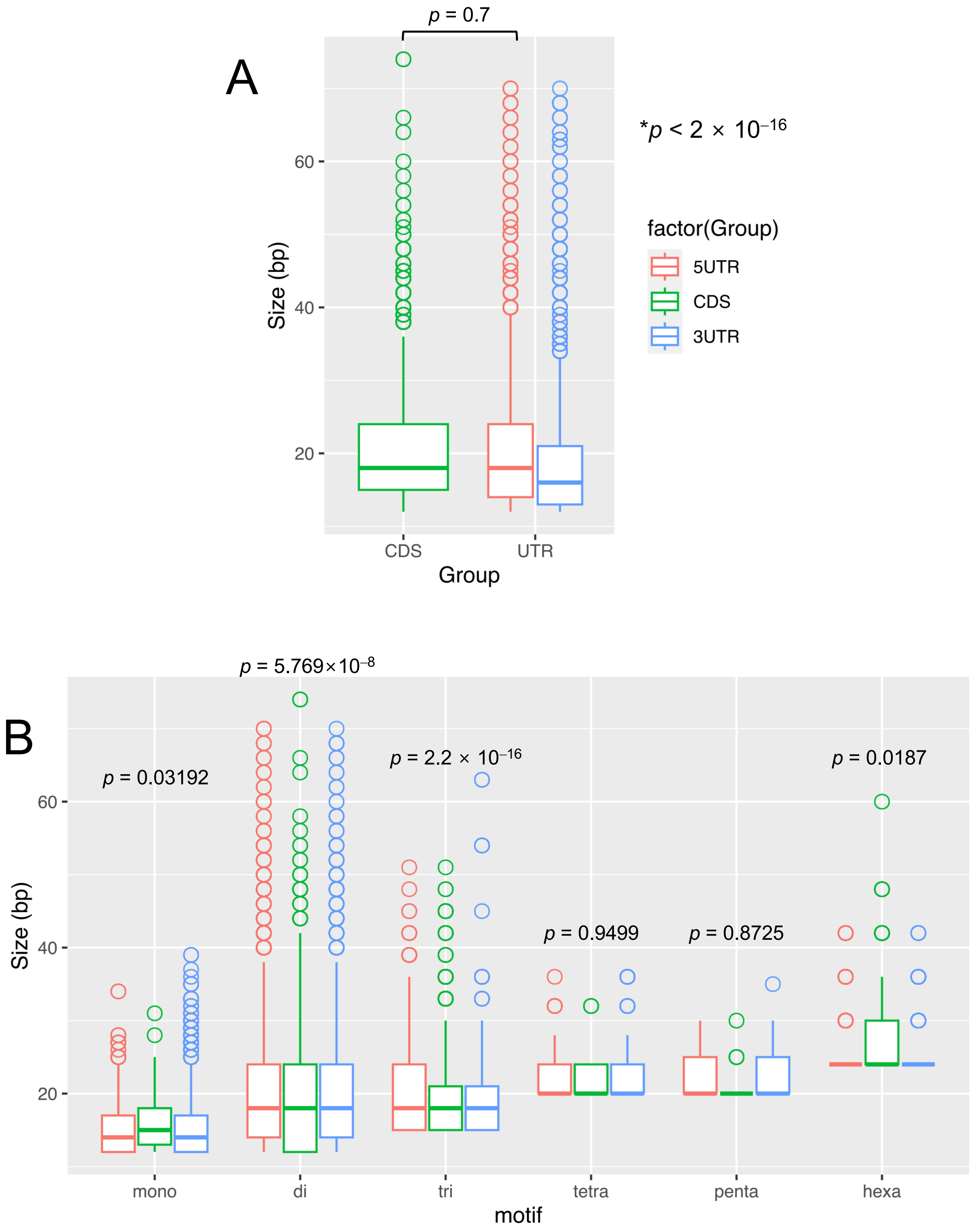

Figure S4. Linkage disequilibrium (LD) map for the 30 transcriptomic SSR markers. Shading is based on the LD test statistic. White boxes indicate marker pairs with significant linkage disequilibrium after Bonferroni correction for 435 pairwise comparisons (adjusted $p < 0.000115$)

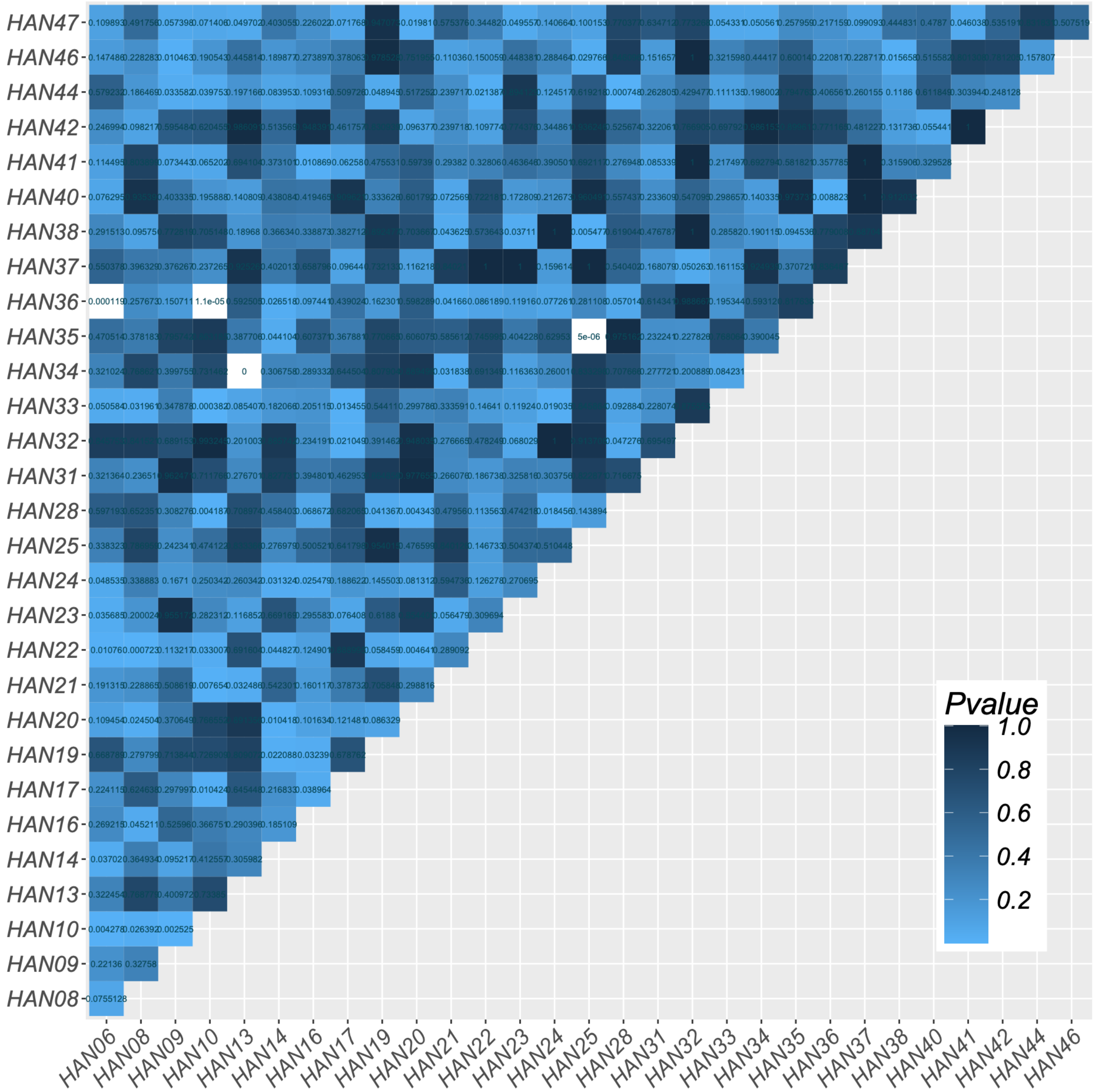

Supplement: Supplementary file 1 — Figures S1–S4: ece373278‐sup‐0001‐Figures.pdf. [file ECE3-16-e73278-s002.pdf]
